# Potential effects of traffic noise on anuran call characteristics in Louisiana, USA during winter

**DOI:** 10.1002/ece3.11679

**Published:** 2024-06-30

**Authors:** Jane M. Kunberger, Ty J. Price, Chloe Crawford, Allison A. Vestal‐Laborde, Ashley M. Long

**Affiliations:** ^1^ Agricultural Center and School of Renewable Natural Resources Louisiana State University Baton Rouge Louisiana USA

**Keywords:** advertisement call, Anura, Cajun chorus frog (*Pseudacris fouquettei)*, cricket frog (*Acris* spp.), Louisiana, urbanization

## Abstract

Urban environments expose wildlife to levels of anthropogenic noise they would not experience in rural areas (e.g., traffic noise), and research suggests that many species adjust their acoustic signals for optimal transmission in urban soundscapes. However, our understanding of anuran (order Anura) responses to noise pollution in urban environments of the southeastern United States is limited, particularly for species that can breed during winter. Our goal was to examine how vocal anuran advertisement call characteristics during winter varied with increasing distance from roadways in bottomland hardwoods of Louisiana, USA. We deployed acoustic recording units at two sites (i.e., rural and urban) perpendicular to Interstate 10 at 200‐, 400‐, and 600‐m intervals (i.e., close, middle, and far) from November 2019 to January 2020. We detected Cajun Chorus Frogs (*Pseudacris fouquettei*) and Cricket Frogs (*Acris* spp.) at our rural site, and only detected Cricket Frogs at our urban site. At the rural site, Cajun Chorus Frogs produced longer duration notes at the far location compared to the middle location. At the urban site, Cricket Frogs produced higher dominant frequency calls at the close location compared to the far and middle locations and longer duration notes at the far location compared to the close location. We were unable to account for additional factors in our models (e.g., temperature, noise levels), but our results generally align with previous research. Our study provides baseline data for future research to examine the potential effects of traffic noise on winter advertisement calls in locations with similar environmental conditions and species.

## Graphical abstract

Our goal was to examine how vocal anuran advertisement call characteristics during winter varied with increasing distance from Interstate 10 (i.e., close, middle, and far locations) at a rural and urban site in Louisiana, USA. We found that Cajun Chorus Frogs (*Pseudacris fouquettei*) and Cricket Frogs (*Acris* spp.) detected at the rural site had few differences in their advertisement call characteristics across locations, but that Cricket Frogs at the urban site had advertisement call characteristics that varied in dominant frequency and duration across locations.

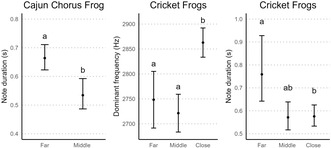

## 1 INTRODUCTION

Urbanization causes widespread habitat loss and fragmentation, which can displace native species and cause local extirpations (Fahrig, 2003; Liu et al., 2016; but see Willmott et al., 2022). Anthropogenic activities can further affect remaining habitat through several mechanisms, including chemical pollution (e.g., road runoff; Dorchin & Shanas, 2010; Gallagher et al., 2014), artificial light (Brain & Anderson, 2020; Pothukuchi, 2021), and anthropogenic noise (Berkhout et al., 2023; Duquette et al., 2021), resulting in consequences to wildlife health, reproductive success, and behavior, among others (Fischer & Lindenmayer, 2007; Heinrichs et al., 2016; Murray et al., 2019). Traffic noise is a common source of anthropogenic noise pollution that can affect a wide range of taxa (Kunc & Schmidt, 2019) and may alter acoustic signaling behaviors and characteristics (Duquette et al., 2021). With anticipated expansions in urban land cover and human populations in the coming years (Chi et al., 2019; Li et al., 2019) and a trend of increasing traffic volume in the southeastern United States (Federal Highway Administration, 2023), it is crucial to understand how factors like traffic noise could affect wildlife in this region.

Anurans (order Anura; i.e., frogs) can be particularly vulnerable to anthropogenic noise, and effects of increased traffic noise can have far‐reaching consequences for anuran behavior and reproductive success. Most male anurans produce advertisement calls to attract mates and defend territories (Gerhardt, 1994; Lee et al., 2023) but traffic noise overlaps many species' frequency niches and has high amplitude (Bee & Swanson, 2007; Cunnington & Fahrig, 2010; Grace & Noss, 2018), which can mask anuran vocalizations (Bee & Swanson, 2007; Lee et al., 2023). To compensate for traffic noise, some species increase the dominant frequency (Caorsi et al., 2017; Grenat et al., 2019; Leon et al., 2019), amplitude (Halfwerk et al., 2016; Leon et al., 2019), call rate (Cunnington & Fahrig, 2010; Grenat et al., 2019; Legett et al., 2020), or number of notes (Grace & Noss, 2018; Leon et al., 2019) of their vocalizations, whereas others avoid calling during periods of high background noise (Vargas‐Salinas & Amézquita, 2013). Though these strategies can improve vocalization audibility, changing call characteristics can be energetically costly, constrained by physiology, and can increase predation risk (Gerhardt, 1994). Furthermore, females can struggle to recognize male vocalizations with call characteristics that differ from the population mean (Bee & Swanson, 2007; McLean et al., 2013; Vargas‐Salinas et al., 2014). Decreased reproductive success near roads with high traffic could lead to localized decreased anuran abundance (Hoskin & Goosem, 2010), changes in morphology (e.g., body size; Hoskin & Goosem, 2010; but see Leon et al., 2019), and decreased occurrence of anuran species with call characteristics that overlap traffic noise (Cosentino et al., 2014).

Though the number of studies on anuran responses to anthropogenic noise has increased in recent years (Jerem & Mathews, 2021), we have limited information on how traffic noise affects anuran vocalizations in the southeastern United States, particularly for species that can breed during the winter months. Anurans that can breed during the winter may benefit from decreased competition for resources (see Ethier et al., 2021). In addition, different abiotic cues can affect winter calling behaviors when compared to anurans that breed during warm weather (Plenderleith et al., 2018; Saenz et al., 2006). With seasonal differences in anuran reproductive behaviors, research investigating the effects of anthropogenic noise during the winter months could help us understand how expanding urbanization might affect anuran reproductive success and demographics throughout the entire year. Our goal was to examine how vocal anuran advertisement call characteristics during winter varied with increasing distance from roadways in bottomland hardwoods of Louisiana, USA. At our site with higher traffic (i.e., the urban site), we predicted that anurans at the location closest to the interstate would have higher dominant call frequencies than anurans of the same species at the location furthest from the interstate to overcome the frequency masking of traffic noise (Caorsi et al., 2017; Grenat et al., 2019; Leon et al., 2019). We also expected that anurans at the location closest to the interstate at the urban site would have shorter note durations than anurans of the same species at the location furthest from the interstate (Zaffaroni‐Caorsi et al., 2023). At our site with lower traffic (i.e., the rural site), we expected to see no differences in advertisement call characteristics across sampling locations.

## 2 MATERIALS AND METHODS

### 2.1 Study site

We collected data in southeast Louisiana from November 2019 to January 2020 at two study sites along Interstate 10—a transcontinental interstate that spans 3959 km from Florida to California, with 441 km located in Louisiana (Federal Highway Administration, 2022). Our study sites were >20 km from one another, with one site in West Baton Rouge Parish, which we called our “rural” site, and the other site in East Baton Rouge Parish, which we called our “urban” site. Our rural site was >1 km from neighborhoods or businesses and was outside of the city limits of Baton Rouge, whereas our urban site was at a university research station with public greenspace that is surrounded by housing developments and businesses and located inside of the city limits of Baton Rouge. Both sites are composed of bottomland hardwood forest and the predominant tree species included White Oak (*Quercus alba*), Green Ash (*Fraxinus pennsylvanica*), American Elm (*Ulmus americana*), Water Oak (*Q. nigra*), Nutall Oak (*Q. texana*), and Sugarberry (*Celtis laevigata*) with an understory of Yaupon (*Ilex vomitoria*), Poison Ivy (*Toxicodendron radicans*), and Chinese Privet (*Ligustrum sinense*).

In 2019, the annual average daily traffic on Interstate 10 was 54,000 vehicles near our rural site and 173,000 vehicles near our urban site (Louisiana Department of Transportation and Development, 2023). Across our entire study period, temperature during recording hours (19:00 to 06:00) ranged from −2.0 to 28.4°C at our rural site and 1.7 to 28.1°C at our urban site. The average within‐night temperature at our rural site was 12.5°C and ranged from 9.6 to 17.6°C. At our urban site, the average within‐night temperature was 15.9°C and ranged from 11.6 to 20.4°C. During our study period, average precipitation was 0.3 cm and average relative humidity was 72% (National Oceanic and Atmospheric Administration, 2023). Our survey locations at both sites experienced minimal disturbance from human activities aside from traffic on the interstate.

### 2.2 Data collection and analysis

At each site, we deployed three Swift Terrestrial Passive Acoustic Recording Units (ARUs; Cornell Lab of Ornithology, Ithaca, New York, USA) perpendicular to the interstate at 200‐, 400‐, and 600‐m intervals (i.e., close, middle, and far). We deployed ARUs 1.8‐m high on trees with microphones facing toward the interstate and set each ARU to record ambient noise between the hours of 19:00 and 06:00 (i.e., sunset to sunrise). We paired one ARU at each site with a REED SD‐4023 Data Logging Sound Level Meter (REED Instruments, Wilmington, NC, USA) to quantify noise levels throughout each night and rotated these devices weekly among locations at each site.

We conducted our analyses in Program R (V. 4.3.1; R Development Core Team, 2023) with packages seastackplot (V. 0.0.1.0; Stuart et al., 2024), Seewave (V. 2.1.6; Sueur et al., 2008), dplyr (V. 1.1.4; Wickham et al., 2023), and ggplot2 (V. 3.4.2; Wickham, 2016), and exported plots with package ggpubr (V. 0.6.0; Kassambara, 2023). We summarized the mean, standard deviation, and range of noise levels for our entire study period and visualized these data using sea stack plots (Stuart et al., 2024). We recorded 948 h of acoustic data during our sampling period and randomly selected 1 min per hour to search for anuran vocalizations within our recordings. We used Seewave to identify anurans calling within each 1‐min segment. When we detected an anuran in a recording, we identified the call to species and used SonoBirdTM (V. 1.5.8; DNDesign, Arcata, CA, USA) to estimate the dominant frequency and note duration for each recording. We defined note duration as the average duration of individual notes within an advertisement call sequence or “bout” (Köhler et al., 2017). We used dominant frequency and note duration in our analyses because these characteristics are less affected by environmental variables like temperature when compared to other advertisement call characteristics (e.g., advertisement call rate, bandwidth; Köhler et al., 2017; Micancin & Wiley, 2014). While three species of Cricket Frogs (i.e., Southern Cricket Frogs [*Acris gryllus*], Northern Cricket Frogs [*A. crepitans*], and Blanchard's Cricket Frogs [*A. blanchardi*]) could have occurred at our study sites, the differences we observed in Cricket Frog dominant frequencies within sites (see below) were less than the differences found between sympatric Cricket Frog species in previous studies (see Micancin & Wiley, 2014). As such, we grouped all Cricket Frog calls as *Acris* spp.

We used linear models to examine the effects of distance from the interstate (i.e., far, middle, or close) on each species' or species group's dominant frequency (Schneider et al., 2010). Because note duration did not follow a normal distribution, we used generalized linear modeling with gamma distributions and inverse‐link functions to examine this variable in relation to distance from the interstate (Nelder & Wedderburn, 1972). We analyzed data from each site separately to minimize the potential for geographic variation in call characteristics to influence our results. Last, we summarized the differences in predicted values across locations for estimates with 95% confidence intervals that did not include 0 (Grueber et al., 2011).

## 3 RESULTS

**FIGURE 1:**
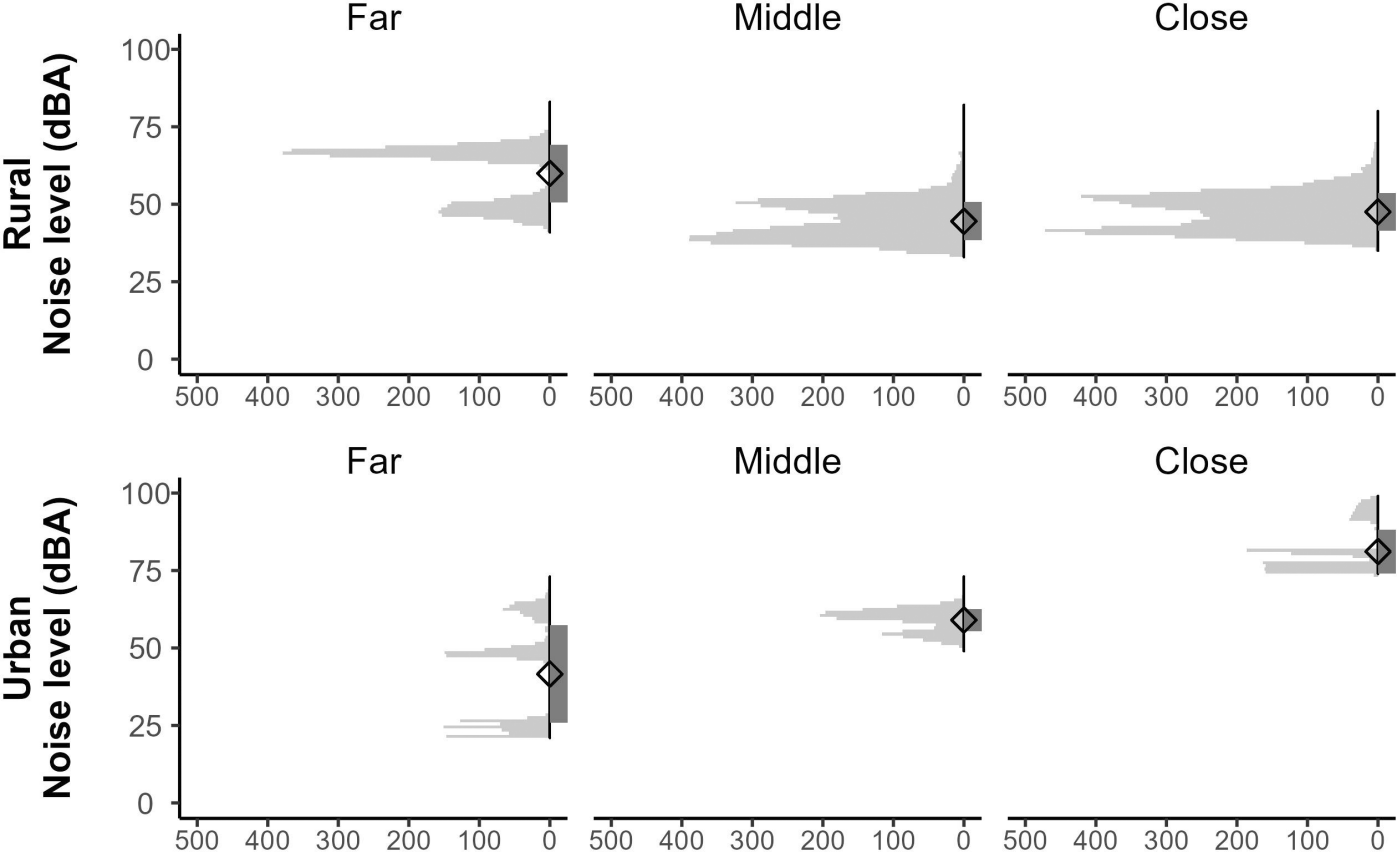
Sea stack plots showing mean (diamonds), SD (gray rectangles), and histograms for noise level observations at study sites representing differing levels of traffic (i.e., rural or urban) and distances to Interstate 10 (i.e., far, middle, or close location) for our study examining the effect of traffic noise on vocal anuran advertisement call characteristics in Louisiana, USA from November 2019 to January 2020.

Noise levels ranged from 33.0 to 82.9 dBA at our rural site and 21.6–98.8 dBA at our urban site (Figure 1). At our rural site, the mean noise level was ~30% greater at the far location than the middle and close locations (Figure 1). In contrast, the mean noise level at our urban site was highest at the close location, with this location having 1.4–2 times greater noise levels than any other location that we sampled during our study (Figure 1). The mean noise level was similar between the rural far and urban middle locations (Figure 1). We collected fewer noise level measurements at the urban site due to logistic constraints (Figure 1), so these measurements may not fully encompass all noise levels experienced at that site throughout the sampling period.

Of the 948 1‐min files that we examined, 74 contained anuran advertisement calls. We detected both Cajun Chorus Frogs (*Pseudacris fouquettei*) and Cricket Frogs at our rural site, and only detected Cricket Frogs at our urban site. In total, we detected Cajun Chorus Frogs in 28 files and Cricket Frogs in 18 files at the rural site, and detected Cricket Frogs in 28 files at the urban site. At our rural site, we detected both Cajun Chorus Frogs and Cricket Frogs at the far and middle locations but did not detect vocal anurans at the close location. In contrast, we detected Cricket Frogs at all locations at the urban site.

**FIGURE 2:**
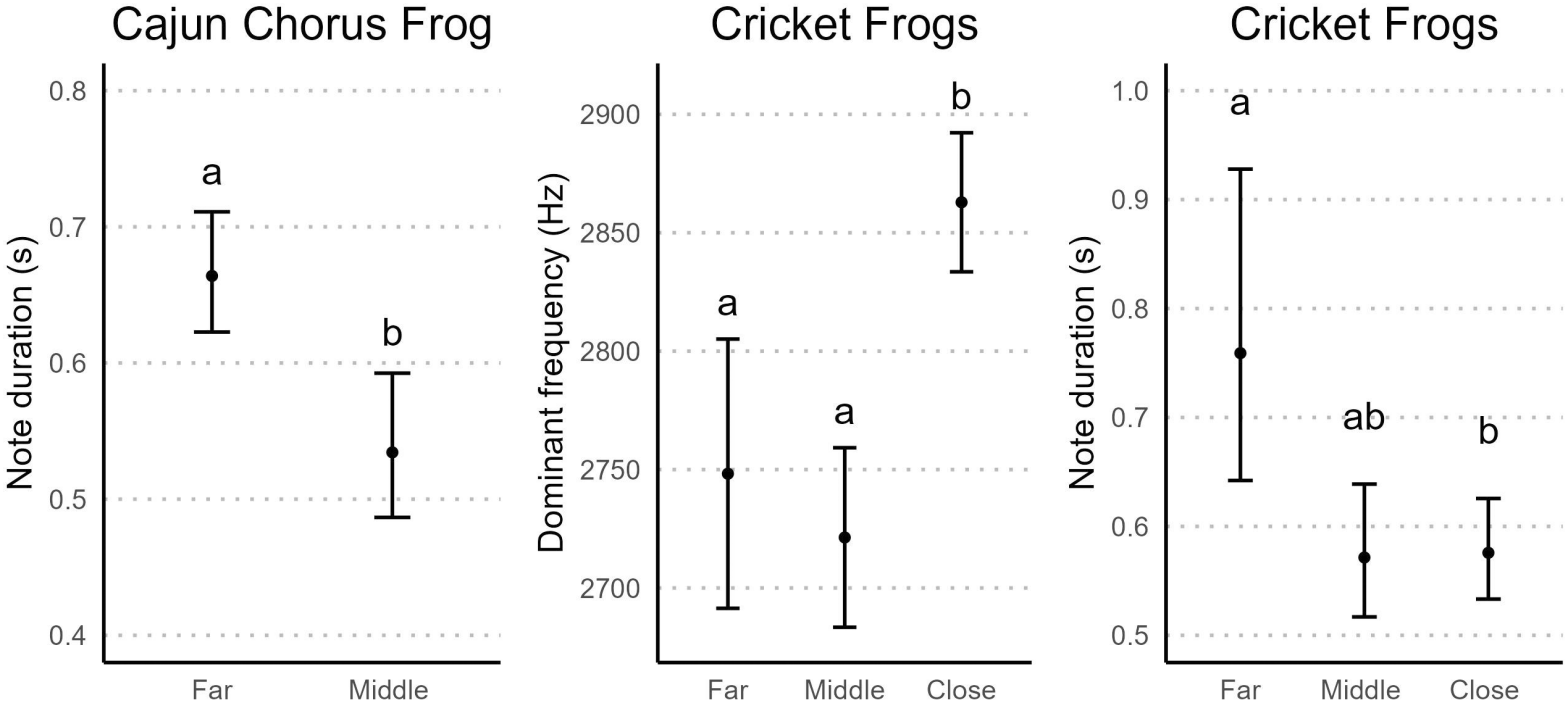
Predicted values and 95% confidence intervals from linear regression (dominant frequency) and generalized linear models (note duration) for Cajun Chorus Frog (*Pseudacris fouquettei*) advertisement call characteristics recorded at our rural site and Cricket Frog (*Acris* spp.) advertisement call characteristics recorded at our urban site in Louisiana, USA from November 2019 to January 2020. Locations represented differing distances to Interstate 10 (i.e., far, middle, or close location). Letters denote significant differences between sites based on model estimates with 95% confidence intervals that did not include 0.

The Cajun Chorus Frog duration model predicted 25% longer duration notes at the far location compared to the middle location at the rural site (Table 1, Figure 2). The Cajun Chorus Frog dominant frequency model had a 95% confidence interval that included 0 for the location parameter, so we considered this model uninformative (Table 1). Similarly, both Cricket Frog models for the rural site had 95% confidence intervals that included 0 for the location parameter (Table 1). At the urban site, the Cricket Frog dominant frequency model predicted an average of 128 Hz higher dominant frequency at the close location compared to the far and middle locations (Table 1, Figure 2). Last, the Cricket Frog duration model for the urban site predicted 32% longer duration notes at the far location compared to the close location (Table 1, Figure 2).

**TABLE 1:** Linear regression (dominant frequency) and generalized linear model (note duration) parameter estimates and 95% confidence intervals for anuran advertisement call characteristics recorded at sites with differing levels of traffic noise (i.e., rural or urban site) and distances to Interstate 10 (i.e., far, middle, or close location) in Louisiana, USA from November 2019 to January 2020. Species included Cajun Chorus Frogs (*Pseudacris fouquettei*), which we only detected at the rural site, and Cricket Frogs (*Acris* spp.).

Species	Metric	Location	Estimate	2.5%	97.5%
Cajun Chorus Frog (*n* = 28)	Dominant frequency	Middle	−4.03	−88.77	80.72
	Note duration	Middle	0.37	0.16	0.57
Cricket Frogs: Rural (*n* = 18)	Dominant frequency	Middle	−97.95	−340.49	144.60
	Note duration	Middle	0.27	−0.04	0.60
Cricket Frogs: Urban (*n* = 28)	Dominant frequency	Far	−114.62	−180.51	−48.73
		Middle	−141.56	−190.92	−92.19
	Note duration	Far	−0.42	−0.68	−0.14
		Middle	0.01	−0.21	0.24

## 4 DISCUSSION

We found few differences in within‐taxa anuran advertisement call characteristics across locations at the rural site, suggesting that lower traffic noise on Interstate 10 had limited masking effects on advertisement calls. Furthermore, because noise levels were similar between the rural close and middle locations, it is unlikely that traffic noise affected our detections of species at the rural close site. Instead, it is possible that our data subset missed vocalizing anurans at the close location or that factors beyond traffic noise (e.g., runoff; Dorchin & Shanas, 2010; Gallagher et al., 2014) could better explain species or species group detections at this location. Last, the higher noise levels we recorded at the rural far location could be attributed to insect noise, which could have caused the increase in Cajun Chorus Frog note duration (Schwartz & Bee, 2013), though we were unable to identify the source of noise at this location.

Traffic noise at the urban close location likely overlapped in frequency with Cricket Frog advertisement calls, resulting in the observed changes in dominant frequency. Increasing advertisement call frequencies can overcome the masking effects of traffic noise (Caorsi et al., 2017; Grenat et al., 2019; Leon et al., 2019), though morphology often limits the extent of these responses, leading to variable success in broadcasting information to conspecifics (Gerhardt, 1994). We also observed shorter note duration at the close location when compared to the far location. Shorter note durations can be related to increases in note rate, which contributes to signal redundancy, thus increasing the probability that conspecifics detect the signal (Köhler et al., 2017; Schwartz & Bee, 2013). Alternatively, high noise levels at the close location could have caused shorter note durations without affecting note rate because the increased energy expenditure associated with increasing note rate outweighed the perceived benefits from an increase in call effort (see Zaffaroni‐Caorsi et al., 2023). However, we did not measure note rate because temperature can affect this characteristic (Köhler et al., 2017) and are therefore limited in our ability to make conclusions about the relationships between traffic noise, note rate, and note duration in our study.

Our results provide baseline information regarding the potential effects of traffic noise on advertisement calls for Louisiana anurans that can breed during the winter months (i.e., Cajun Chorus Frogs can breed from November to April; Cricket Frogs can breed year‐round; Boundy & Carr, 2017; Dundee & Rossman, 1989). To date, few studies have used passive acoustic monitoring to study anuran responses to anthropogenic noise during the winter. Because many anuran call characteristics are related to environmental conditions (e.g., temperature, anuran chorusing; Köhler et al., 2017), understanding the range of effects of anthropogenic noise on advertisement calls requires information on anuran responses throughout the entire year, particularly for anurans that can breed during several seasons. However, our limited number of observations may not represent the full scope of advertisement call characteristics for Cajun Chorus Frogs and Cricket Frogs across locations. Furthermore, we grouped Cricket Frogs and assumed we consistently detected one species due to the relatively small differences in dominant frequency across locations (see Micancin, 2008; Micancin & Wiley, 2014), though it is possible that we detected multiple species at the urban site, confounding observed differences in call characteristics across species with the effects of traffic noise. In addition, both temperature and fluctuations in background noise (e.g., traffic noise) can affect anuran vocalizations (Gerhardt, 1994; Lee et al., 2023), but we were unable to incorporate temperature and traffic noise directly into our analyses of anuran call characteristics because we did not collect these data. As such, we limited our analyses to dominant frequency and note duration, which are less affected by temperature (Köhler et al., 2017), and summarized noise levels at our sampling locations. We had a limited sample of noise levels at our urban site and factors outside of traffic noise (e.g., insects) could have affected our recordings; however, noise levels were highest at the urban close location and decreased with increasing distance from the interstate, so it is likely that interstate traffic contributed to these observations. Last, we chose not to collect vegetation measurements at sampling locations because the composition was similar across locations and there was no leaf cover or herbaceous understory during our winter study, though differences in vegetative cover, litter, and access to freshwater could have led to differences in species occurrence or abundance (e.g., Bomske & Bickford, 2019; Pitt et al., 2017) and, thus, differences in our estimations of advertisement call characteristics at each location.

In recent years, scientists have noted a precipitous decline in many anuran populations due to the disease Chytridiomycosis (caused by the fungus *Batrachochytrium dendrobatidis*; Skerratt et al., 2007), which can cause both lethal and sub‐lethal effects in larval and adult anurans (Bielby et al., 2015; Urbina et al., 2021). In addition to this disease, pollution in waterways (e.g., non‐native leaf litter, metals; Cotten et al., 2012; Hayden et al., 2015), reproductive interference from non‐native anuran species (Kraus, 2015; Tennessen et al., 2016), and habitat loss and fragmentation (Covarrubias et al., 2021) have likely contributed to anuran population declines. Furthermore, for anurans that breed during the winter, changes in winter temperatures related to climate change could have significant effects on the timing and duration of reproduction (Plenderleith et al., 2018; Saenz et al., 2006). When combined with these threats, urbanization and associated increases in traffic noise could have extensive effects on anurans. Our findings for Cricket Frogs aligned with those of other anurans (e.g., Caorsi et al., 2017; Grenat et al., 2019; Leon et al., 2019), but changes in advertisement call characteristics in response to traffic are species‐specific and often affected by a myriad of factors like species' frequency ranges, amplitudes, and mate‐attracting behaviors (e.g., chorusing), as well as the frequency range and noise levels of nearby traffic (Caorsi et al., 2017; Cunnington & Fahrig, 2010; Grenat et al., 2019). Furthermore, intraspecific variation in characteristics like body size and condition can affect an individual's adaptations to anthropogenically altered soundscapes (Harding et al., 2019) and could result in different acoustic responses to traffic noise across individuals. Factors beyond noise, like substrate vibrations associated with anthropogenic activities, can also affect advertisement call characteristics (Caorsi et al., 2019). Because of the variation in anuran responses to traffic noise, it is crucial that researchers in the southeastern United States continue to study the effects of rapidly urbanizing environments on anuran acoustic signaling behaviors across seasons, which combined with other threats could have far‐reaching consequences to anuran reproductive success, community demographics, and, ultimately, survival.

## AUTHOR CONTRIBUTIONS

**Jane M. Kunberger:** Data curation (lead); methodology (equal); writing – original draft (lead); writing – review and editing (equal). **Ty J. Price:** Conceptualization (lead); data curation (supporting); funding acquisition (lead); investigation (lead); methodology (equal); writing – original draft (supporting); writing – review and editing (supporting). **Chloe Crawford:** Methodology (equal); writing – review and editing (supporting). **Allison A. Vestal‐Laborde:** Writing – review and editing (supporting). **Ashley M. Long:** Conceptualization (lead); funding acquisition (lead); methodology (lead); project administration (lead); supervision (lead); writing – original draft (supporting); writing – review and editing (equal).

## 5 FUNDING INFORMATION

McIntire‐Stennis Cooperative Forestry capacity funding program (project no. LAB94479) from the USDA National Institute of Food and Agriculture.

## CONFLICT OF INTEREST STATEMENT

The authors declare no conflicts of interest.

## Supporting information


Data S1.

## Data Availability

The data that support the findings of this study are available in the Supporting Information of this article.
